# Comprehensive Review on Plant Cytochrome P450 Evolution: Copy Number, Diversity, and Motif Analysis From Chlorophyta to Dicotyledoneae

**DOI:** 10.1093/gbe/evae240

**Published:** 2024-11-07

**Authors:** Yuanpeng Fang, Zheng Tai, Keyi Hu, Lingfeng Luo, Sanwei Yang, Mengmeng Liu, Xin Xie

**Affiliations:** College of Agriculture, Guizhou University, Guiyang 550025, PR China; College of Agriculture, Guizhou University, Guiyang 550025, PR China; College of Agriculture, Guizhou University, Guiyang 550025, PR China; College of Agriculture, Guizhou University, Guiyang 550025, PR China; College of Agriculture, Guizhou University, Guiyang 550025, PR China; College of Agriculture, Guizhou University, Guiyang 550025, PR China; College of Agriculture, Guizhou University, Guiyang 550025, PR China

**Keywords:** cytochrome P450 evolution, plant diversity, gene duplication, motif analysis

## Abstract

Cytochrome P450 enzymes (CYPs) are widely distributed among various plant groups and constitute approximately 1% of the total number of protein-coding genes. Extensive studies suggest that CYPs are involved in nearly all molecular processes that occur in plants. Over the past two decades, the identification of CYP genes has expanded rapidly, with more than 40,000 CYP genes and 819 CYP families being discovered. Copy number variation is a significant evolutionary characteristic of gene families, yet a systematic characterization of the copy evolution patterns in plant CYP gene families has been lacking, resulting in confusion and challenges in understanding CYP functions. To address these concerns, this review provides comprehensive statistics and analyses of the copy number and diversity of almost all plant CYP gene families, focusing on CYP evolution from Chlorophyta to Dicotyledoneae. Additionally, we examined the subfamily characteristics of certain CYP families with restricted copy changes and identified several CYP subfamilies that play pivotal roles in this event. Furthermore, we analyzed the structural conservation of CYPs across different taxa and compiled a comprehensive database to support plant CYP studies. Our analysis revealed differences in the six core conserved motifs of plant CYP proteins among various clans and plant taxa, while demonstrating similar conservation patterns for the ERR (glutamic acid-arginine-arginine) triad motifs. These findings will significantly facilitate the understanding of plant CYP gene evolution and metabolic diversity and serve as a valuable reference for researchers studying CYP enzymes.

**Significance**

This review comprehensively analyzes the evolution and diversity of plant cytochrome P450 enzymes (CYPs), focusing on copy number variations and evolution patterns from Chlorophyta to Dicotyledoneae. Identifying key subfamilies and highlighting structural conservation across taxa, the study offers valuable insights into plant *CYP* gene evolution and metabolic diversity. These findings will advance research on CYP enzymes, providing a crucial reference for researchers in the field.

## Introduction

Cytochrome P450 (CYP) was initially discovered in rat livers in 1958 as a protein that facilitates the oxidative transformation of hydrophobic molecules, increasing their hydrophilicity (Klingenberg 1958). The term “cytochrome P450” was formally coined by Omura and Sato in 1962, who identified this as terminal oxidase, a member of a protein class characterized by carbon monoxide binding in the reduced state, absorption peak at 450 nm, and containing a heme group (Omura and Sato 1962). The CYP gene family has been observed in all organisms, ranging from noncellular entities such as viruses to prokaryotes (archaea and bacteria) and eukaryotes (plants, animals, and fungi) (Omura 1999; Lamb et al. 2019; Teng et al. 2019; Ngcobo et al. 2023; Rieseberg et al. 2024).

Studying plant CYPs and identifying their substrates becomes particularly challenging when utilizing the Basic Local Alignment Search Tool (BLAST) to compare sequences with <20% identity. This limitation hinders accurate inference of evolutionary relationships and functional similarities among plant CYPs (Zhang et al. 2020; Hansen et al. 2021). To address this issue, Dhabalia Ashok et al. (2024) introduced sequence similarity networks to identify other and potential relationships, and Dadras et al. (2023) determined that CYP diversity interferes with metabolic diversity. Nonetheless, CYPs share a common 3D structure, consisting primarily of α-helices and a smaller proportion of β-folds (Zhang et al. 2020; Hansen et al. 2021). Furthermore, multiple structural motifs within CYP enzymes remain conserved, influencing their subcellular localization and catalytic activity (Wang et al. 2018; Liu et al. 2023). Additionally, studies have demonstrated that copy number variations within gene families can significantly impact an organism's adaptive capacity to specific environments (Li et al. 2015). Plant CYPomes (the total number of CYP genes in a given species) are generally more extensive when compared with those found in animals or microorganisms (Sello et al. 2015; Khumalo et al. 2020). For instance, the green algae *Chlamydomonas reinhardtii* (Chlorophyta) includes at least 39 CYP genes (Nelson 2006; Nelson et al. 2008), while 98 CYP genes have been identified in *Physcomitrella patens* (Bryophyta) (Nelson et al. 2008). In many angiosperms, the total number of genes encoding CYPs can exceed 1% of protein-coding genes (Nelson 2018). Analyzing structural and copy number variations could provide valuable insights into the evolution of plant CYPs. However, the absence of a comprehensive overview currently impedes CYP research.

Despite several reviews on plant CYPs (Omura 1999; Werck-Reichhart and Feyereisen 2000; Hansen et al. 2021), most have overlooked the genome-wide variation in copy number of these enzymes. These reviews generally offer a qualitative assessment of the presence or absence of different CYP enzyme classes, without considering quantitative changes in their abundance (Hansen et al. 2021) or providing a comprehensive understanding of their evolution. Furthermore, the absence of effective research tools has contributed to challenges in studying plant CYPs and their evolution, including phylogenetic and structural variations.

To address these limitations, this comprehensive overview aims to provide recent insights into the genome- and transcriptome-wide identification of plant CYP families. We discuss the molecular characteristics of CYP copy number evolution and sequence diversity, focusing on specific examples of various biosynthetic pathways affected by copy number variations. Additionally, we summarize the structural conservation of core motifs across different taxa. Lastly, we compile a list of supporting databases for plant CYP research. By consolidating these findings, this review paper significantly contributes to the understanding of plant CYP evolution and metabolic diversity. It serves as a valuable introductory reference for researchers interested in studying plant CYP enzymes.

### Emergence of Plant CYPs

#### Number of CYPs

In a recent report on the number of CYP genes, Nelson's laboratory revealed that a vast collection of more than 300,000 CYP protein sequences has been compiled from various species. Among these, over 41,000 CYP sequences have been accurately defined according to established nomenclature (Nelson 2018). This extensive knowledge encompasses CYP sequences from viruses, aquatic animals, plants, fungi, bacteria, and archaea (Nelson 2018; Ershov et al. 2019; Lamb et al. 2019; Ngcobo et al. 2023). Nelson (2018) hypothesized that with the advancement of platforms such as the Genome 10K project, i5k, and the Global Invertebrate Genomics Alliance (GIGA), the assembly of over one million cytochrome P450 sequences would be achievable after 2020.

Current classification efforts, based on CYP sequence alignments, have made steady progress. By 2020, at least 3,204 CYP families had been identified in fungal genomes, making them the most diverse group, followed by bacteria, protists, and animals, each with at least 1,000 CYP families. In plants, 819 CYP families have been named, while oomycetes, archaea, and viruses have fewer than 35 named CYP families (Sello et al. 2015; Khumalo et al. 2020; Table 1). Although plants possess a rich variety of secondary metabolites, the number of named CYP gene families appears relatively limited and insufficient compared with that of fungi or animals. To gain further insights into the evolutionary role of CYPs during plant development, it is crucial to assess their homologous CYP sequences using high-quality genomes.

**Table 1:** Diversity of CYP genes in the life tree (Sello et al. 2015; Khumalo et al. 2020)

Species category	Species subcategory	P450 count	P450 families
Animals	Mammals	4,558	18
	Other vertebrates	3,268	19
	Insects	22,173	1,031
	Noninsect invertebrates	7,150	880
Plants	*–*	42,102	819
Fungi	*–*	28,260	3,204
Chromista	*–*	356	15
Protozoa	*–*	5,807	1,374
Bacteria	*–*	17,236	1,910
Archaea	*–*	1,204	34
Viruses	*–*	37	13
Total	*–*	132,151	9,317

#### Number of Genome-Level Plant Cytochrome P450s

To provide a comprehensive understanding of the evolutionary history of CYPs, we collected data from plant genome-level identification studies. We excluded studies that lacked a valid CYP gene family classification or had incomplete classifications, such as those on pepper (*Capsicum annuum*), cotton (*Gossypium hirsutum*), ginseng (*Panax ginseng*), and thunderclap (*Tripterygium wilfordii*) (Wang et al. 2018; Kato et al. 2020; Sun et al. 2021). We compiled data from 36 plant CYP identification studies conducted at the genome-wide level, including 1 systematic CYP taxonomy for Chlorophyta and 1 for Bryophyta, 5 studies focused on Poales Monocotyledons (specifically, Poaceae), and 29 studies on Dicotyledoneae (spanning 18 orders) (Table 2). Integration of genome data revealed that ancient algae had accumulated a substantial number of CYP gene copies and underwent selective retention, gene loss, and sequence variation. These processes contributed to the accumulation of CYP genes, resulting in the observed diversity in plants. Chlorophyta exhibited a relatively large number of CYP genes, with 39 copies (Nelson 2006; Nelson et al. 2008), while Bryophyta showed a slight expansion, with 98 copies, representing a 2.51-fold increase compared with that of Chlorophyta (Nelson 2006; Nelson et al. 2008). These events partly explain the differential selection of polyploidy and metabolic diversity in the common ancestor of Chlorophyta and Bryophyta (Devos et al. 2016; Table 2). A clear evolutionary pattern has been observed from Monocotyledons to Dicotyledoneae (angiosperms) (Angiosperm Phylogeny Group et al. 2016). Poaceae, a diverse group of Monocotyledons, demonstrates conserved CYP clans among five plant species. However, the extent of sequence accumulation varies significantly, with the number of CYP genes in Panicinae appearing independent of copy events. The polyploid *Saccharum spontaneum* has a higher number compared with that of the diploid *Sorghum bicolor*. Duplication events have not resulted in significant CYP accumulation (∼10%), whereas tandem repeat events appear to have impacted CYP accumulation, resulting in an approximate 10% decrease in the *Zea mays* genome. When comparing *Oryza sativa* with *Triticum aestivum*, clear differences in copy number accumulation were observed in the polyploid *T. aestivum*, with up to a 3.88-fold increase (Nelson et al. 2008; Li and Wei 2020; Table 2). However, further analysis is needed to examine the reported CYP copy numbers in many species, particularly in Monocotyledons where complex evolutionary processes are likely involved. A dataset of 29 CYPs for Dicotyledoneae indicated the loss of certain clans (74 and 711), while clan 727 may be present in some species. The lowest number of available CYP copies (76) was recorded in *Utricularia gibba* (Tubiflorae), whereas *Helianthus annuus* (Asterales) has the highest copy number at 462 (Chen et al. 2020; Li et al. 2023; Table 2). The significant discrepancies in CYP copy numbers across different species highlight the need for further research before drawing definitive conclusions.

**Table 2:** Number of plant *CYP* genes determined at the genome level

Taxon	Species	51	74	97	710	711	727	71	72	85	86	746	4	741	ULT	Total	References
C-Volvocales	*Chlamydomonas reinhardtii*	1	0	4	1	12	0	0	0	8	0	1	2	4	6	39	Nelson (2006), Nelson et al. (2008)
B-Eubryales	*Physcomitrella patens*	1	3	3	2	0	1	58	6	8	15	1	0	0	0	98	Nelson et al. (2008)
M-Poales	*Saccharum spontaneum*	12	4	3	1	6	1	243	45	37	38	0	0	0	0	390	Nelson (2019)
M-Poales	*Sorghum bicolor*	15	4	3	1	4	1	201	49	34	38	0	0	0	0	350	Fang et al. (2021)
M-Poales	*Zea mays*	6	6	3	1	4	1	168	56	29	40	0	0	0	0	314	Liu et al. (2018)
M-Poales	*Triticum aestivum*	37	15	12	7	12	3	781	152	97	172	0	0	0	0	1288	Li and Wei (2020)
M-Poales	*Oryza sativa*	10	4	3	4	5	1	190	36	37	42	0	0	0	0	332	Nelson et al. (2008), Wei and Chen (2018)
D-Asterales	*Helianthus annuus*	1	6	3	2	2	0	275	56	62	55	0	0	0	0	462	Chen et al. (2020)
D-Asterales	*Lactuca sativa*	1	7	3	1	1	0	230	36	46	49	0	0	0	0	374	Chen et al. (2020)
D-Apiales	*Panax notoginseng*	2	4	3	1	1	0	102	21	28	26	0	0	0	0	188	Xiong et al. (2021)
D-Sapindales	*Citrus clementina*	1	3	3	1	2	1	194	23	33	23	0	0	0	0	284	Liu et al. (2023)
D-Solanales	*Solanum lycopersicum*	1	7	2	1	1	0	126	30	30	24	0	0	0	0	222	Vasav and Barvkar (2019)
D-Malpighiales	*Ricinus communis*	1	3	3	1	1	1	120	20	36	21	0	0	0	0	207	Kumar et al. (2013)
D-Myrtales	*Aquilaria agallocha*	1	3	3	0	1	0	84	7	23	16	0	0	0	0	138	Das et al. (2022)
D-Brassicales	*Arabidopsis thaliana*	2	2	3	4	1	0	167	23	29	33	0	0	0	0	264	Li and Wei (2020)
D-Brassicales	*Brassica rapa*	3	2	2	3	1	0	211	34	40	58	0	0	0	0	354	Yu et al. (2017)
D-Brassicales	*Brassica oleracea*	5	2	6	2	1	0	207	22	38	60	0	0	0	0	343	Yu et al. (2017)
D-Brassicales	*Carica papaya*	1	2	3	1	1	1	71	14	34	15	0	0	0	0	143	Nelson et al. (2008)
D-Contortae	*Fraxinus excelsior*	1	2	4	1	1	0	51	12	16	13	0	0	0	0	101	Li et al. (2023)
D-Contortae	*Olea europaea*	1	2	6	1	1	0	55	11	17	16	0	0	0	0	110	Li et al. (2023)
D-Caryophyllales	*Fagopyrum tataricum*	1	0	3	1	2	0	173	24	37	44	0	0	0	0	285	Sun et al. (2020)
D-Fagales	*Populus trichocarpa*	2	6	3	1	2	2	162	34	62	36	0	0	0	0	310	Nelson et al. (2008)
D-Lamiales	*Salvia miltiorrhiza*	1	2	2	1	1	0	60	11	17	14	0	0	0	0	109	Li et al. (2023)
D-Lamiales	*Pogostemon cablin*	2	2	3	1	1	0	84	15	16	18	0	0	0	0	142	Li et al. (2023)
D-Malpighiales	*Linum usitatissimum*	2	10	5	1	2	2	190	35	45	42	0	0	0	0	334	Babu et al. (2013)
D-Rosales	*Medicago sativas*	1	4	4	1	2	0	88	12	19	26	0	0	0	0	157	Liao et al. (2017)
D-Rosales	*Glycine max*	2	6	5	2	4	1	195	32	50	35	0	0	0	0	332	Guttikonda et al. (2010)
D-Rhoeadales	*Eschscholzia californica*	1	6	1	1	0	0	119	5	15	41	0	0	0	0	189	Hori et al. (2018)
D-Sapindales	*Citrus sinensis*	1	0	3	1	2	0	131	15	33	16	0	0	0	0	202	Mittapelli et al. (2014)
D-Sapindales	*dh Citrus sinensis*	1	3	3	1	2	0	188	22	33	19	0	0	0	0	272	Mittapelli et al. (2014)
D-Solanales	*Solanum tuberosum*	1	2	2	1	1	0	59	10	15	16	0	0	0	0	107	Li et al. (2023)
D-Tubiflorae	*Utricularia gibba*	1	1	2	1	1	0	40	10	8	12	0	0	0	0	76	Li et al. (2023)
D-Tubiflorae	*Mimulus guttatus*	1	2	3	1	1	0	50	10	15	15	0	0	0	0	98	Li et al. (2023)
D-Urticales	*Morus notabilis*	1	3	3	1	0	1	98	19	26	22	0	0	0	0	174	Ma et al. (2014)
D-Vitales	*Vitis vinifera*	2	6	2	1	1	0	134	32	34	23	0	0	0	0	235	Jiu et al. (2020)

D, Dicotyledoneae; M, Monocotyledons; C, Chlorophyta; G, Gymnospermae; B, Bryophyta; ULT, uncertain lateral transfer.

#### Number of Transcriptome-Level Plant CYPs

We collected data on the number of CYPs identified at the plant transcriptome level to tentatively infer the evolutionary processes driven by the diversity of plant secondary metabolites. We excluded studies that did not provide effective or complete classifications of CYP gene families. Our collection of published data on CYP identification at the transcriptome level included systematic classification data for two Gymnospermae (Cupressales and Pinales) and six Dicotyledoneae (Asterales, Apiales, Dipsacales, Lamiales, and Rosales). Transcriptome assemblies often contain fragmented sequences, which may result in a lower number of copies compared with the actual state. However, they can still be used to infer gene copy evolution, as demonstrated by studies on *Salvia miltiorrhiza* CYP gene copy numbers (Chen et al. 2014; Yang et al. 2022). Gymnospermae represents a sister branch of Angiospermae, and the characterization of their CYP copies can provide insights into the evolutionary origin of Angiospermae genes and selective retention strategies in their large genomes (Xiong et al. 2021). The CYP copies in Gymnospermae indicate the accumulation of at least 100 copies, a number comparable to that of Bryophyta, suggesting that the whole-genome duplication event shared by Gymnospermae and Angiospermae did not significantly impact CYP number expansion. In contrast, all six studies on Dicotyledoneae report copy numbers well above 100, further raising doubts about the current assessments of CYP gene copy numbers (Tables 2 and  3).

**Table 3:** Number of plant *CYP* genes determined at the transcriptome level

Taxon	Species	51	74	97	710	711	727	71	72	85	86	Total	References
G-Cupressales	*Taxus chinensis*	1	3	2	1	0	0	52	9	37	13	118	Liao et al. (2017)
G-Pinales	*Picea glauca*	1	5	4	1	1	1	47	3	17	23	103	Chen et al. (2014), Warren et al. (2015)
D-Asterales	*Jacobaea vulgaris*	3	7	3	1	1	0	144	27	15	20	221	Chen et al. (2020)
D-Asterales	*Jacobaea aquatica*	4	4	3	1	1	0	96	20	12	16	157	Chen et al. (2020)
D-Dipsacales	*Lonicera japonica*	2	5	3	1	1	1	71	28	26	13	151	Qi et al. (2017)
D-Apiales	*Aralia elata*	1	4	3	1	1	0	79	18	23	20	150	Warren et al. (2015)
D-Lamiales	*Salvia miltiorrhiza*	1	0	3	0	1	1	65	16	14	13	114	Yang et al. (2022)
D-Rosales	*Cajanus cajan*	1	4	3	1	2	1	131	28	33	22	226	Cheng et al. (2020)

D, Dicotyledoneae; M, Monocotyledons; C, Chlorophyta; G, Gymnospermae; B, Bryophyta; ULT, uncertain lateral transfer.

#### CYP Diversity

**Fig. 1.**
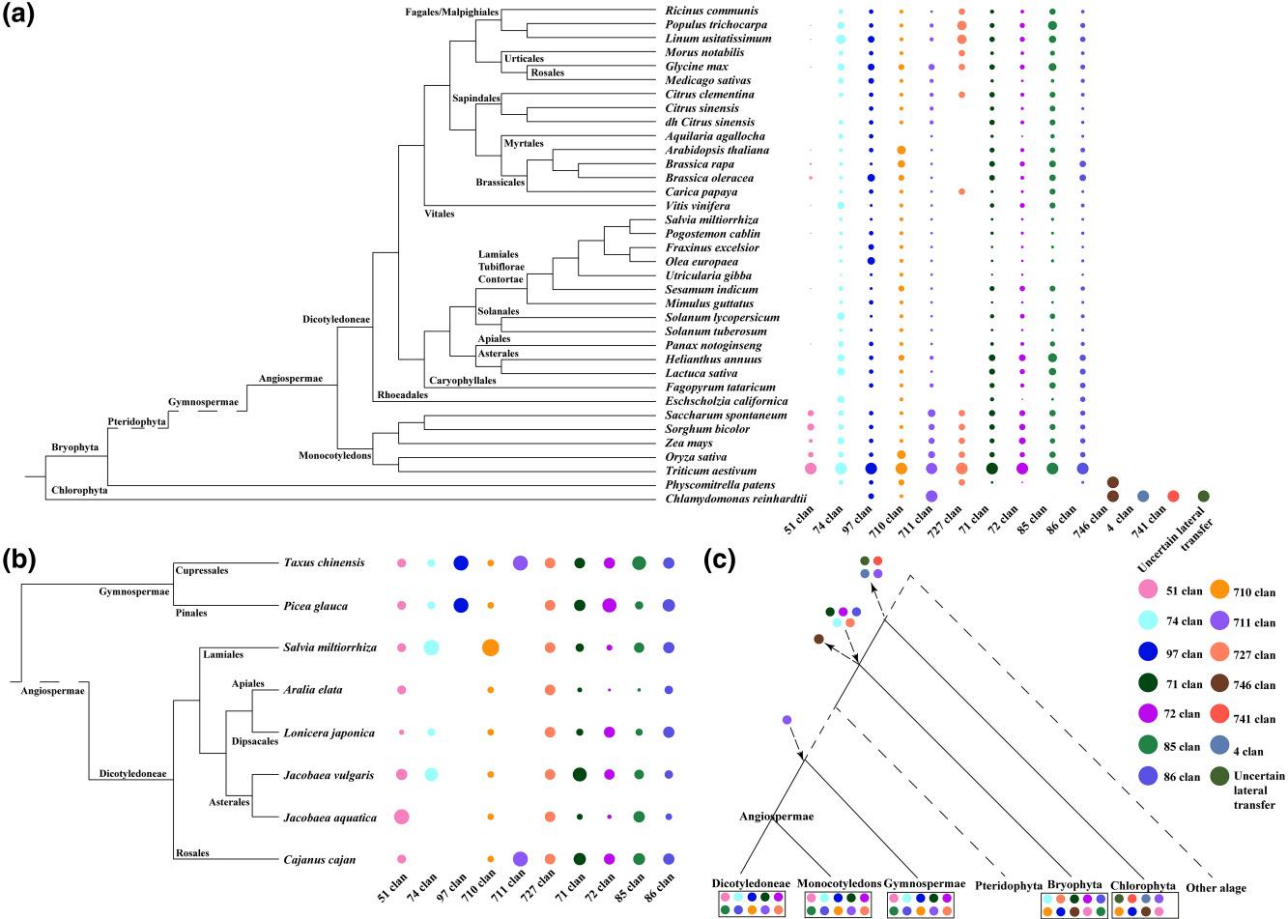
Whole-transcriptome data on cytochrome P450 (CYP) families of plants and distribution numbers of clan. Number of gene copies at the clan level resulted from the identification of CYP gene families. The analysis was carried out using the angiosperm phylogeny group (APG) system a) at the genome and b) transcriptome levels. c) Clan formation and maintenance in the main evolutionary clades of plants.

**Fig. 2.**
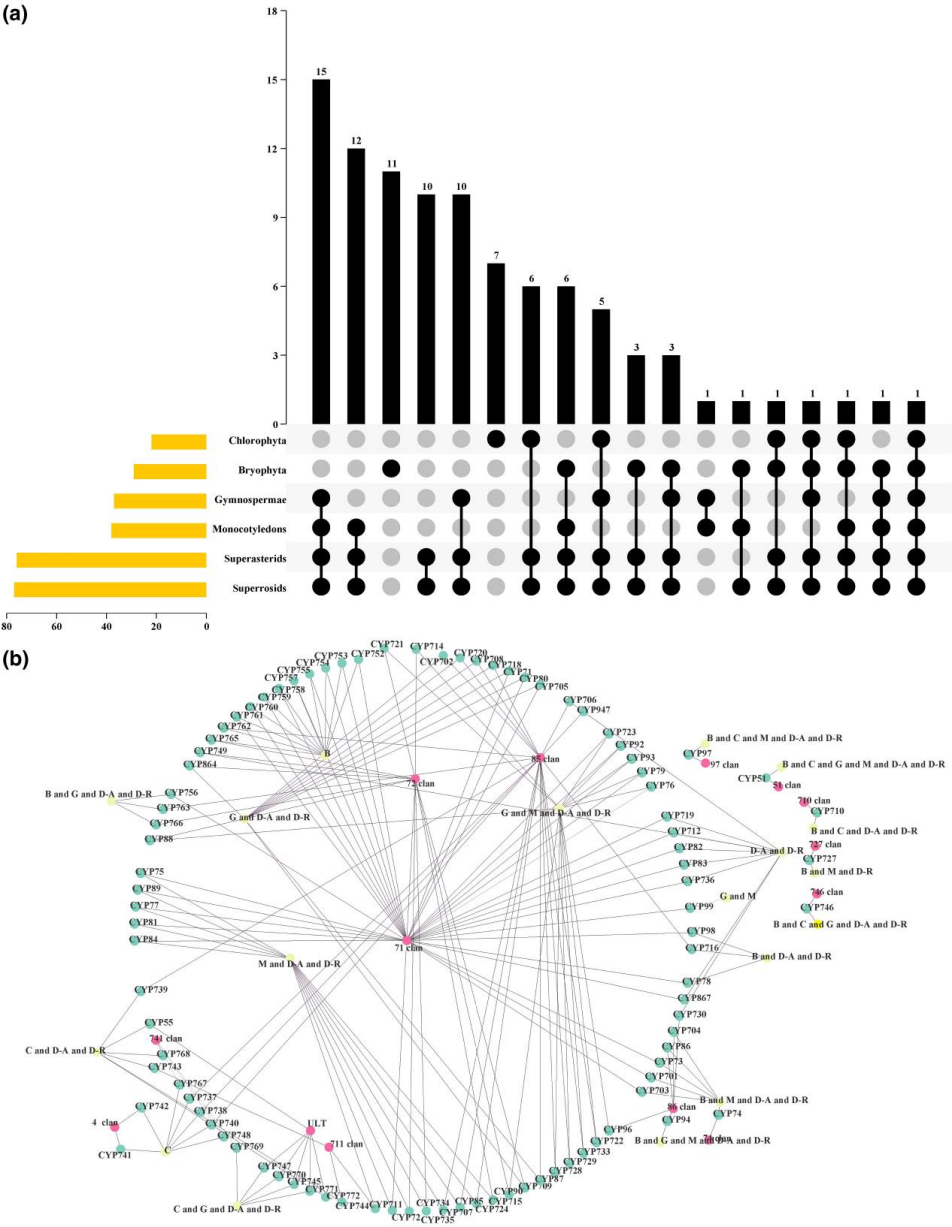
Cytochrome P450 (CYP) family distribution in plant taxa. a) UpSet diagrams of CYP families identified in different taxa; the plot shows intersections among six taxa. b) Distribution of CYP family clans and class groups plotted using Cytoscape 3.9.1. D-A, superasterids; D-R, superrosids; M, Monocotyledons; C, Chlorophyta; G, Gymnospermae; B, Bryophyta; ULT, uncertain lateral transfer.

**Fig. 3.**
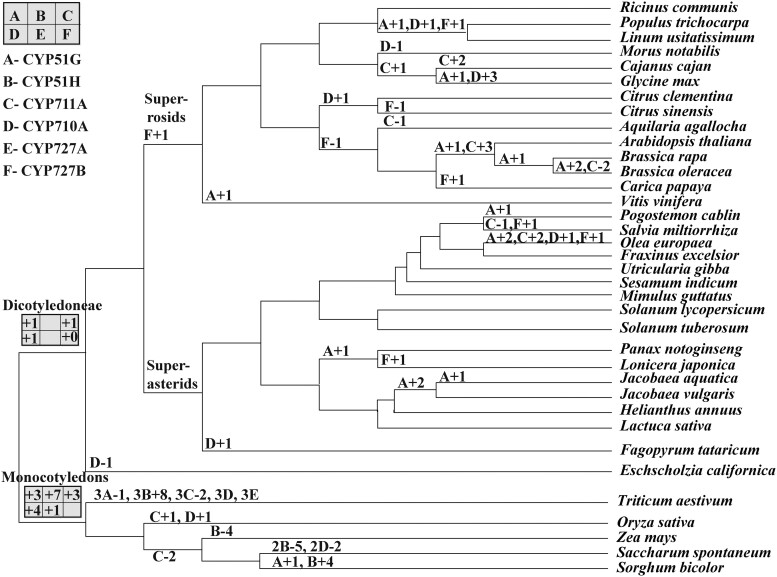
Copy number changes of cytochrome P450 (CYP) families in four single-family groups of angiosperms.

To address the extensive number of CYP copies and the wide range of substrate selection, Nelson et al. (1996, 2009) established a classification system based on sequence homology. This system employs a multilevel marker, where the name consists of CYP + number (indicating family) + letter (indicating subfamily) + number. The inclusion of clan-level distributions based on phylogenetic relationships has greatly facilitated the study of CYP function and speculation on metabolic diversity (Nelson 2018). CYP genes in plants are classified into two categories based on function or structure: type A (specialized metabolic processes, primarily within clan 71) and nontype A (basal metabolic processes) (Li and Wei 2020). Typical plants mainly belong to Chlorophyta, which includes three monophyletic taxa (Chlorophyta, Streptophyta, and Prasinodermophyta), among which Streptophyta has the highest species diversity and can be divided into Angiospermae, Gymnospermae, Pteridophyta, and Bryophyta (Fang et al. 2022). Although many branches are not included in this analysis, the CYP gene distributions at the clan, family, and subfamily levels were characterized for 44 plant species across Chlorophyta and non-Pteridophyta Streptophyta clades (Figs. 1–3).

#### Clan Diversity of Plant CYPs

Chlorophyta represents a monophyletic taxon that includes land plants within the green algae group; *Chlamydomonas reinhardtii*, a typical Chlorophyta species, serves as a model for understanding the potential origin of land plant genes (Misumi et al. 2008). In *C. reinhardtii*, we observed a significant accumulation of CYP genes, including clans 746, 4, and 741, and uncertain lateral transfer (ULT). Clan 711 showed the highest presence in *C. reinhardtii* (Fig. 1a; Li and Wei 2020). Plant terrestrialization has been started on land since the formation of Embryophytes, and Bryophytes may be at the base of this process, with the exception of some Charophyceae, for which *P. patens* is tentatively used to denote a major innovation in the process (Rensing et al. 2020). In terms of clan distribution, *P. patens* lost clans 4 and 741, and ULT, but retained clan 711, similar to *C. reinhardtii*. Moreover, there was an expansion of various clans, including clans 71, 72, 86, 74, and 727 (Fig. 1a and c; Tables 2 and 3; Li and Wei 2020).

Moving from the common ancestor of Bryophytes and Gymnosperms to the seed plants (gymnosperms and angiosperms), clan 746 was lost, and the clan 10 system became predominant. Gymnospermae exhibited the presence of clans 51, 71, 72, 85, 86, 74, 97, 710, 711, and 727, which were further maintained in Angiospermae (Nelson et al. 2008; Liao et al. 2017). Further analysis revealed inconsistent accumulation of different clan gene copies in different branches. Clan 51 showed significant abundance in Poaceae, likely due to the expansion of CYP51H. Clan 711 was found to be abundant in Poaceae, while clan 710 was abundant in Brassicales (Fig. 1; Tables 2 and 3). These observations suggest a complex and selective accumulation of CYP genes in plants, but further investigation is needed to understand the exact evolutionary mechanisms involved.

#### Family Diversity of Plant CYPs

The previous section provided initial explanations for the evident diversity observed in different taxa (Fig. 1; supplementary fig. S1, Supplementary Material online; supplementary tables S1 and S2, Supplementary Material online). However, a detailed examination of the patterns of copy retention is necessary. We investigated the formation of family diversity in CYP sequences and observed that the six core taxa (Superasteridae, Superrosidae, Monocotyledons, Gymnospermae, Bryophyta, and Chlorophyta) exhibit abundant selective retention mechanisms. Figure 2a illustrates that only a small number of CYP families have been consistently retained during the evolution of Chlorophyta or Bryophyta, while Gymnospermae have retained a larger number of the same gene families (up to 15) throughout evolution. Monocotyledons appear to have innovatively produced 12 CYP families. In Dicotyledoneae (superasterids and superrosids), ten CYP families may have emerged; no significant differences at the CYP family level were observed between superasterids and superrosids, further supporting their close genetic relationship (Fig. 2a; supplementary table S3, Supplementary Material online; Angiosperm Phylogeny Group et al. 2016).

A total of 37 CYP families are missing from monophyletic taxa, and their reappearance in nonmonophyletic taxa has persisted throughout plant evolution, suggesting many selective processes for many CYPs (Fig. 2a). The abundance of CYP families led to their retention in only a limited number of taxa, such as Bryophyta, which retained 7 and 11 CYP families, and CYP99 (clan 71), which is present only in Monocotyledons and gymnosperm taxa. These findings contribute, to some extent, to the molecular basis of metabolic diversity in different taxa (Fig. 2; Lamb and Waterman 2013; Nelson 2018; Hansen et al. 2021). Furthermore, although three clans are conserved across all five major taxa, only the CYP51 family of clan 51 is conserved at the family level (Figs. 1 and 2b). Clan 711 was lost during plant evolution, primarily owing to the loss of CYP743 and CYP744 and the robust presence of clan 711 in angiosperms, from which CYP711 is derived (Figs. 1 and 2b).

#### Subfamily Differentiation of CYPs in Angiosperms

In addition to the diversity of CYPs, angiosperms exhibit rich variations in copy numbers. A direct correlation between the expansion patterns of certain monophyletic clans and the differentiation of their subfamilies has been proposed (Fang et al. 2021). To assess the contribution of subfamilies to the accumulation of CYP copy numbers, we conducted a survey focusing on four major monophyletic clans: CYP51 and CYP711, which show abundant accumulation in Gramineae (Poaceae), and CYP710 and CYP727, which exhibit selective accumulation in Dicotyledoneae (Figs. 1 and 2; supplementary fig. S2, Supplementary Material online; supplementary table S4, Supplementary Material online).

#### Restricted Expansion of CYP51

CYP51G is involved in the biosynthesis of membrane sterols, specifically obtusifoliol, which further promotes the synthesis of various steroidal saponins and alkaloids (Michellod et al. 2023; Yin et al. 2023). The CYP51 family is conserved across a wide range of organisms, including fungi and animals, with an expansion from bacterial origins but with some sequence variations (Lepesheva and Waterman 2007; Lamb et al. 2021). The typical clan 51 consists solely of the CYP51 family, and clear taxonomic differences have been observed within this branch among angiosperms.

The CYP51 family in Poaceae (grass family) exhibits the largest expansion in copy numbers (Fig. 1), and only two subfamilies, CYP51G and CYP51H, are present in angiosperms. Among them, CYP51G is the oldest plant CYP51 subfamily gene, and a single copy is retained in all nonangiosperms (Fig. 1). The copies of *CYP51G1* differ significantly between Dicotyledoneae and Monocotyledons, with Monocotyledons possessing three copies and Dicotyledoneae having a single copy of *CYP51G1* (Fig. 3). However, owing to the limited number of studies on CYPs in Monocotyledons, only relevant data for CYP51G from Poaceae are currently available (Liu et al. 2018; Nelson 2019; Li and Wei 2020; Fang et al. 2021). Further confirmation is required to determine if this copy number is fully representative of Monocotyledons.

Moreover, specific expansions and contractions of CYP51G have been observed in multiple species. For example, there is a single gene deficiency in the hexaploid *T. aestivum* (wheat), while the diploid *S. bicolor* (sorghum) possesses only three copies of *CYP51G1* and does not undergo polyploid expansions (Nelson 2019; Li and Wei 2020; Fig. 3). Expansions of CYP51G genes are present in taxa such as Brassicaceae (Brassica and Jacobaea), *Vitis vinifera* (grapevine), *S. bicolor*, *Pogostemon cablin*, and *Glycine max* (soybean) (Fig. 3; Yu et al. 2017; Chen et al. 2020; Jiu et al. 2020; Fang et al. 2021).

A novel subfamily, CYP51H, has evolved exclusively in Poaceae, and these genes may be the primary source of expansion within clan 51. The CYP51H subfamily is involved in the synthesis of antimicrobial triterpene glycosides, acts as a 14α-demethylase, and is involved in the synthesis of phytosterols and brassinosteroids (Kunii et al. 2012; Jiao et al. 2022). The copy number of CYP51H in Poaceae is likely to be seven, and it is accompanied by a triplication event in hexaploid *T. aestivum*, resulting in the expansion of eight additional copies (Fig. 3). *Sorghum bicolor* and *Z. mays* each possess a single copy of CYP51H (Fig. 3), which may contribute to significant expansion and enrichment of metabolites in the CYP gene repertoire of *S. bicolor* (Fang et al. 2021). Finally, the tetraploid *S. spontaneum* also exhibits selective retention of CYP51H, with a reduction of five copies in addition to the duplication event (Fig. 3; Nelson 2019).

#### Restricted Extension of CYP711

Plant CYP711As, also known as MAX1, play a role in the oxidation of intermediate carbolactone in trihydroxylactone biosynthesis (Zhang et al. 2014; Jia et al. 2018; Vinde et al. 2022). Most functionally characterized CYP711As convert caratone to caratonic acid, unipodal lactone, and 4-deoxyobanol, ultimately leading to hydroxylation into orobanchol. The genes belonging to clan 711 exhibit possible deficiencies or local expansions in angiosperms and are the predominant CYP family in this group (Figs. 1 and 2).

The copy number of *CYP711A1* in nonflowering plants is either 0 or 1, while angiosperms generally maintain stable copies of CYP711 (Fig. 1). The CYP711A subfamily, similar to CYP51G, shows significant variations in copy size among taxa (Fig. 3). Notably, there are genetic variants in this subfamily, potentially leading to a two-gene deficiency in hexaploid *T. aestivum* (Li and Wei 2020). In superasterids, there is minimal variation in the CYP711A subfamily, with *CYP711A1* predominantly existing as a single copy. In contrast, superrosids exhibit expansions of the CYP711A subfamily without clear taxonomic selection (Fig. 3). While CYP711A is lost in *S. miltiorrhiza* and *Aquilaria agallocha*, the functional implications of this phenomenon remain unknown, such as whether it indicates a lower diversity of metabolites or the presence of compensatory enzymes.

#### Restricted Extension of CYP710

Plant CYP710A is a sterol C-22 desaturase involved in driving the diversity of plant membrane sterols (Morikawa et al. 2006). This gene family, along with its functional homologs in fungi, is derived from CYP61, with the CYP710A and CYP710B subfamilies representing the homologous subfamilies of streptophytes and green algae, respectively (Hansen et al. 2021). Clan 710 shows some expansion, with only the CYP710A subfamily present in angiosperms (Fig. 1).

In Monocotyledons, the copy number of CYP710A has expanded up to four times that of Dicotyledoneae, with most species retaining canonical basal copy numbers (Fig. 3). However, the tetraploid *S. spontaneum* may exhibit deficiencies in two genes (Nelson 2019), while the model plant rice contains an additional copy of CYP710A (Wei and Chen 2018). *Eschscholzia californica* and *Morus notabilis* have lost all copies of CYP710A (Ma et al. 2014). Some species, including *G. max*, *Fagopyrum tataricum*, *Olea europaea*, and Citrus, may possess copies of CYP710A (Fig. 3; Mittapelli et al. 2014; Sun et al. 2020; Liu et al. 2023). In conclusion, these studies suggest a stable copy accumulation of CYP710A with notable exceptions in a few species, the underlying causes of which require further investigation.

#### Large Loss of CYP727

Clan 727 represents the tenth CYP branch in angiosperms and solely comprises CYP727 (Liu et al. 2023). The specific catalytic processes in which CYP727 participates have not been fully verified. Figure 1 indicates that clan 727 is widely extended in angiosperms. In Monocotyledons, a single subfamily of the CYP727 family exists, known as CYP727A, with single copies present in all Poaceae plants, including the tetraploid *S. spontaneum* (Nelson 2019). Interestingly, the hexaploid *T. aestivum* contains three pairs of CYP727A gene copies (Fig. 3; Li and Wei 2020). This strongly suggests a more complex accumulation of CYP727A genes under polyploidy mechanisms. Among Dicotyledoneae, there is only a single CYP727 subfamily, known as CYP727B, and certain species exhibit deletions of this subfamily (Fig. 3). However, selective taxa, including *S. miltiorrhiza*, *O. europaea*, and *Lonicera japonica*, still retain CYP727B copies (Fig. 3).

#### Structure of Plant CYPs

The sequence homology among members of the CYP family from different organisms is generally low, with as little as 20% identical sequences. Even between highly homologous CYP members, significant differences in amino acid composition have been observed (Werck-Reichhart and Feyereisen 2000; Chadha et al. 2018; Wybouw et al. 2019; Calla 2021). Initial studies identified a similar heme-binding loop in all CYPs, which consists of an irregular helix, two α-helices (J and K helices), and two sets of β-folds (Hasemann et al. 1995; Sirim et al. 2010). These structures encompass six conserved motifs: the (P/I)PGPX(P/G)XP motif in the proline-rich region, residue WXXXR in the C-terminal helix, the I-helix (A/G)GX(E/D)T(T/S), the ERR (glutamic acid-arginine-arginine) triad (consisting of the proline-glutaminc acid-arginine-phenylalanine [PERF] residue in the electron transport channel and EXXR residue in the K-helix), and the heme-binding loop FXXGXXXCXG (Chadha et al. 2018; Ershov et al. 2019; Zhang et al. 2020). All CYP sequences contain a conserved P450 structural domain associated with catalysis. Previous studies have shown that while conserved regions of CYP sequences are abundant in different species, the six core motifs exhibit a high rate of change and loss (Fang et al. 2021; Liu et al. 2023). To assess the stability of these motifs, we examined the most conserved elements in 10 CYPs and identified 122,708 occurrences of these motifs, with 6 conserved motifs being highly prevalent (supplementary fig. S3, Supplementary Material online; supplementary tables S5 and S6, Supplementary Material online).

#### Conserved ERR Triad

**Fig. 4.**
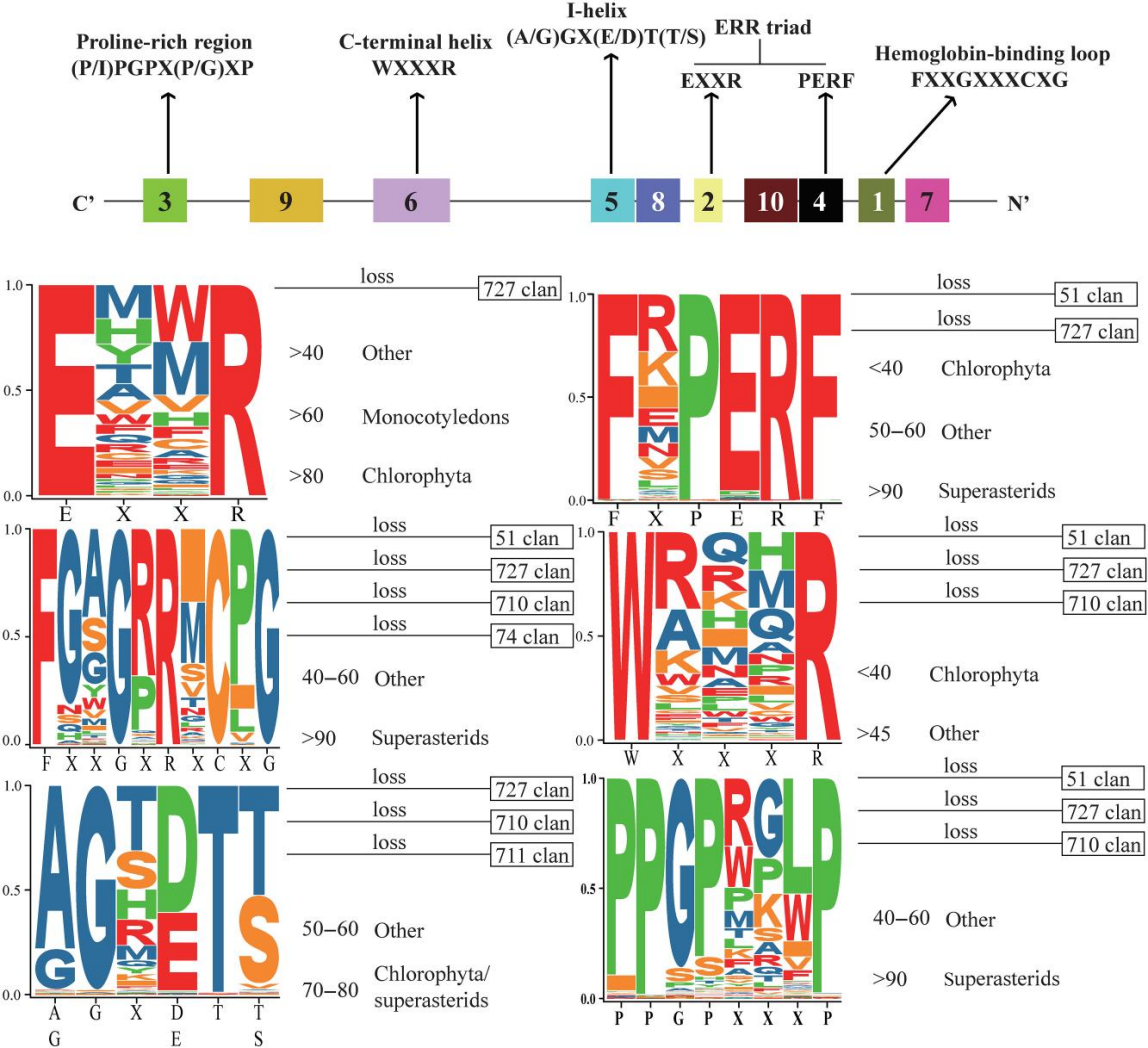
Analyses of plant cytochrome P450 enzymes (CYPs) and WebLogos of six core plant cytochrome P450 (CYP) protein motifs. Proportional distribution and analysis of missing features in the six core motifs are shown.

The ERR triad comprises two core structures, the K-helix motif (EXXR) and the electron transport channel (PERF). The E and R residues of the K-helix and R residue of PERF form an E–R–R ternary structure that helps stabilize the 3D structure of CYP (Hasemann et al. 1995). The EXXR motif is the most conserved motif and is generally absent only in clan 727 (Fig. 4; supplementary fig. S4, Supplementary Material online). Interestingly, the EXXR motif is highly retained in Chlorophyta, followed by Monocotyledons and other plant taxa (Fig. 4). This observation suggests that the diversity of CYPs comes at the expense of stability in their original 3D structure. For example, in *Solanum lycopersicum*, the EXXR motif in the K-helix region is relatively stable, mainly consisting of E(T/S/V/A)(L/F/M)R (Vasav and Barvkar 2019). However, in *Clonorchis sinensis*, this motif is distributed across all CYP types, and multiple EXXR motifs are present in the same sequence (Liu et al. 2023). Different families or subfamilies in the CYP51, CYP710, and CYP711 clan branches exhibit clear motif conservation with rich site variability. For instance, the core motifs of CYP51A/G and CYP51H are stable as EQ(F/H)R, while the variable residues in CYP710A and CYP710B show rich variations in CA-QY (supplementary fig. S4, Supplementary Material online).

The E residue in PERF is generally conserved, although some variation exists at the initial site (phenylalanine-any amino acid-leucine-proline-glutamate-phenylalanine [FXPERF]) (Fig. 4). Similar patterns have been observed in *S. lycopersicum* and *S. bicolor* (Vasav and Barvkar 2019), but the E and F sites appear to be more variable (Vasav and Barvkar 2019; Fang et al. 2021). FXPERF is present in most superasterids but less prevalent in Chlorophyta (Fig. 4). Approximately 83.7% of the sequences of the superasterid *C. sinensis* exhibit variations, although FXPERF is generally well-distributed (Liu et al. 2023). Different families or subfamilies in the CYP74, CYP97, CYP710, and CYP711 clan branches show clear motif conservation, but variability may still exist among members (supplementary fig. S5, Supplementary Material online).

#### Heme-Binding Region

The core catalytic center of CYPs is FXXGXRXCXG, which constitutes the heme-binding motif. The cysteine (C) residue serves as the axial ligand for thiolate-heme binding (Hasemann et al. 1995). The FXXGXRXCXG motif is highly conserved in superasterids, with approximately 99.0% of sequences in the superasterid *C. sinensis* retaining this motif (Liu et al. 2023). However, this motif is largely absent in clans 710, 727, 74, and 51 in different taxa (Fig. 4). Clans 727 and 74 in both *C. sinensis* and *S. bicolor* lack the FXXGXRXCXG motif (Fang et al. 2021; Liu et al. 2023). There is a significant structural variation in the FXXGXRXCXG motif among families and subfamilies, with specific variations observed within groups (supplementary fig. S6, Supplementary Material online).

#### Spiral Conservation

Multiple helical regions of CYPs contain conserved motifs that contribute to structural specificity. These include the C-terminal helix (WXXXR) and I-helix (A/G)GX(E/D)T(T/S). The W and R residues in the C-terminal helix interact with the propionate side chain of heme (Hasemann et al. 1995). As the W and R residues are highly conserved, this motif is less retained in Chlorophyta (Fig. 4). The C-terminal helix is highly conserved in approximately 69.7% of sequences in *C. sinensis* (Liu et al. 2023). However, it is largely absent in clans 710, 727, and 51 across different taxa (Fig. 4); in *C. sinensis*, it is absent in clans 97, 72, 727, 711, 710, 51, and 74 (Liu et al. 2023). The WXXXR motif exhibits variations in different subfamilies or families, with variations specific to certain groups (supplementary fig. S7, Supplementary Material online).

The I-helix region is crucial for oxygen binding and activation in CYPs, and the (A/G)GX(E/D)T(T/S) motif is generally conserved. However, some variation exists at multiple sites (Fig. 4), and in *S. lycopersicum*, the T residue is often replaced by S (Vasav and Barvkar 2019). The (A/G)GX(E/D)T(T/S) motif is retained in only 50% to 60% of sequences in most species, but it appears multiple times in a significant number of sequences in *C. sinensis* CYPs (Liu et al. 2023). It is frequently lost in clans 727, 710, and 711 (Fig. 4), as well as in clans 727 and 74 in *C. sinensis* and *S. bicolor* CYPs (Fang et al. 2021; Liu et al. 2023). The (A/G)GX(E/D)T(T/S) motif shows clear conservation in clans 51, 711, 74, and 97 among different families and subfamilies, but may exhibit variability (supplementary fig. S8, Supplementary Material online).

#### Conservation of Proline-Enriched Regions

The presence of a proline-enriched region in CYPs is believed to be crucial for membrane localization. The core motif of (P/I)PGPX(P/G)XP was represented as PPGPXXXP in this analysis (Fig. 4). Proline-enriched regions exhibit significant differences between species, with higher prevalence in superasterids compared with that of plants from other taxa (Fig. 4). Approximately 90.5% of sequences from the superasterid *C. sinensis* retain this motif, particularly in clan 727 (Liu et al. 2023). However, the motif is absent in clans 710, 727, and 51 in different taxa (Fig. 4); in *C. sinensis*, it is also absent in clan 97 (Liu et al. 2023). The PPGPXXXP motif displays significant variability among different subfamilies or families, with specific variations observed within certain groups (supplementary fig. S9, Supplementary Material online).

### Database Resources for Plant CYPs

Plant CYPs have received relatively less research attention, primarily owing to their high diversity and extensive functional differentiation among homologous genes, which presents significant challenges for researchers (Table 4). To address this issue and facilitate CYP research, Nelson (2009) established a committee for CYP nomenclature and subsequently developed a comprehensive naming database for CYPs across multiple organisms. This resource has played a crucial role in organizing and categorizing CYPs, providing researchers with a valuable tool to navigate the complex landscape of CYPs in various species.

**Table 4:** Database resources for plant cytochrome P450

Name	Website	Year	Function	References
CYPedia	https://bmcplantbiol.biomedcentral.com/articles/10.1186/1471-2229-8-47	2008	Functional research tools	Ehlting et al. (2008)
MPOD	http://medicinalplants.ynau.edu.cn/	2022		He et al. (2022)
CYPED	https://cyped.biocatnet.de/	2007	Instrumental database for sequence similarity matching and family assignment	Fischer et al. (2007), Sirim et al. (2009, 2010), Gricman et al. (2015)
FCFD	http://p450.riceblast.snu.ac.kr/	2008		Park et al. (2008)
Cytochrome P450 Homepage	https://drnelson.uthsc.edu/	2009		Nelson (2009)
Plant P450 Database	https://erda.dk/public/vgrid/PlantP450/	2021	Database for predicting and storing substrates and structures of CYP	Hansen et al. (2021)
PCPD	http://p450.biodesign.ac.cn/	2021		Wang et al. (2021)
P450Rdb	https://www.cellknowledge.com.cn/p450rdb/index.html	2023		Zhang et al. (2024)

### Resources for the Source and Expression of Plant CYPs

#### CYPedia

Ehlting et al. (2008) developed a pioneering database for plant CYP sequences and expression resources, specifically focusing on *Arabidopsis thaliana*. They utilized public Affymetrix ATH1 microarray expression data to establish correlations between CYP gene expression and various factors such as *Arabidopsis* organs and tissues, stress responses, hormone responses, and mutants (Ehlting et al. 2008). This database, known as CYPedia—Cytochrome P450 Expression in Arabidopsis (u-strasbg.fr), provides researchers with access to comprehensive information on CYP genes and their expression patterns in *Arabidopsis*.

#### Multi-Omics Database for Medicinal Plants (MPOD)

He et al. (2022) developed the largest genomic database, MPOD, specifically designed for medicinal plants, focusing on genes associated with potential medicinal compounds, including cytochrome P450 (CYP) genes. This database offers comprehensive expression data for 24 medicinal plants, enabling researchers to investigate CYP gene expression patterns. MPOD provides essential information such as accession numbers, gene lengths, sequences, and reaction equations for 159 CYP genes, with specific emphasis on six medicinal plant CYP genes (*Cyclocarya paliurus*, *Gynostemma pentaphyllum*, *Hemsleya chinensis*, *Neoalsomitra integrifoliola*, *Panax notoginseng*, and *P. vietnamensis*). Researchers can utilize various tools within MPOD, such as Pearson correlation analysis, BLAST support, heatmap generation, phylogenetic trees, primer design, and co-expression analysis, to further analyze plant CYP genes and their functional characteristics.

MPOD represents the most extensive genomic resource available for studying CYP genes in medicinal plants and provides researchers with a range of essential tools for investigating CYP functions. However, it should be noted that MPOD does not offer support for CYP nomenclature, and direct information on expression levels and reference CYP sequence sets is not available on the website. The MPOD database can be accessed at http://medicinalplants.ynau.edu.cn/tools/expression.

### Database Resources for Plant CYP Nomenclature

#### Cytochrome P450 Engineering Database

Fischer et al. (2007) developed the first CYP naming tool, known as CYtochrome P450 Engineering Database (CYPED), primarily for comparing protein sequences and structures within the CYP family. The initial version integrated the sequences of 3,911 proteins and the structures of 25 proteins. Moreover, it utilized the Nelson classification system to classify proteins into homologous families and subfamilies.

Sirim et al. (2009) updated CYPED, incorporating the sequences of 8,613 proteins and the structures of 47 proteins. This update further improved the annotation of functionally and structurally relevant residues. Based on the data from CYPED, the Cytochrome P450 Knowledge Base (CPK) was established, and CYP protein sequences from CYPED were included to provide information on potential substrates and inhibitors (Sirim et al. 2009). In 2010, they subsequently added 31 structures to create reliable hidden Markov models (HMMs), which greatly facilitated the analysis of CYP substrate-residue interactions and interaction sites with redox chaperones. Gricman et al. (2015) further updated CYPED, expanding its capacity to include 16,732 CYP sequences, and subsequently introduced a sequence-based text mining algorithm to CYPED, further enhancing its capabilities (Gricman et al. 2015).

CYPED offers several advantages, including (i) being one of the largest publicly available databases of CYP sequences; (ii) providing annotations on functionally relevant residues, potential substrates, and inhibitors; and (iii) supporting multiple sequence comparisons, phylogenetic trees, and HMM queries. However, it is important to note that (i) CYPED has not been regularly updated since 2015, (ii) it does not specifically focus on plant CYPs, and (iii) the nomenclature used in the database may not be easily understood.

It is now available at https://cyped.biocatnet.de/.

#### Fungal Cytochrome P450 Database

Park et al. (2008) developed the first CYP nomenclature tool specifically designed for fungi, known as Fungal Cytochrome P450 Database (FCFD). This database includes information on 23 potential CYPs of Viridiplantae along with their corresponding sequences, totaling 6,668 sequences. The sequences were preliminarily categorized using Nelson's classification system, allowing for classification into homologous families and subfamilies. The FCFD database offers a user-friendly interface and presents CYP enzyme information in an easily understandable manner. However, it should be noted that FCFD is not a dedicated plant CYP nomenclature database, and the availability of plant-derived CYP data is limited. The FCFD database can be accessed at http://p450.riceblast.snu.ac.kr/.

#### Cytochrome P450 Homepage

Nelson first introduced the Cytochrome P450 Homepage, a comprehensive repository for CYP nomenclature and sequence information, in February 1995; the platform includes nomenclature information for 11,512 CYPs. The Cytochrome P450 Homepage was regularly updated and maintained until 2011; however, no significant updates have been made since then. Regardless, the Cytochrome P450 Homepage is considered the most authoritative tool for CYP nomenclature and provides easy access to the P450 BLAST server sequences. Additionally, Nelson made a large number of named sequences publicly available, facilitating researchers in performing local naming for large-scale CYP sequences. However, as the Cytochrome P450 Homepage is not up-to-date, the inclusion of plant CYP data may not be comprehensive. The Cytochrome P450 Homepage can be accessed at https://drnelson.uthsc.edu/.

### Database Resources for Plant CYP Structures

#### Plant P450 Database

Hansen et al. (2021) curated a comprehensive collection of plant cytochrome P450 (CYP) sequences with well-defined substrates from the existing literature, leading to the development of the Plant P450 Database. This database serves as a reliable repository and storage platform for plant CYP research. It encompasses 874 publicly available plant CYP sequences, along with 7 crystal structures of plant CYPs and 38 plant CYP sequences associated with herbicide metabolism.

The Plant P450 Database stands out as the most trustworthy and up-to-date collection of plant CYP sequences as of 2021. It provides researchers with the most extensive and detailed information regarding plant CYP structures and their involvement in herbicide metabolism. However, it is important to note that the database does not include unstudied CYPs or CYP sequences that lack sufficient homology. Therefore, it should be utilized primarily as a valuable reference tool for CYP functional studies. To access the Plant P450 Database, please visit https://erda.dk/public/vgrid/PlantP450/.

#### Plant Cytochrome P450 Database

Wang et al. (2021) developed the Plant Cytochrome P450 Database (PCPD) specifically for supporting structure prediction and virtual screening of plant CYP sequences. This database includes a collection of 181 plant CYP sequences, along with their functions and corresponding Protein Data Bank (PDB) structures. The plant cytochrome P450 comparative modelling (PCPCM) section of PCPD utilizes AlphaFold2 to optimize the structures of plant CYPs, providing highly accurate predictions within a short timeframe of 30 min. The plant cytochrome P450 ligand docking (PCPLD) section offers an analytical ligand docking tool for virtual screening of plant CYPs.

PCPD is the first integrated sequence-structure docking platform designed for plant CYP proteins and represents an efficient resource for CYP protein structure prediction. However, it should be noted that PCPD contains a limited number of plant CYP sequences and may not cover all types of CYPs. The structural quality of the predicted structures in PCPD also needs to be further validated. Additionally, the ligand library in PCPD is currently small and may not be comprehensive enough to infer substrate predictions for all CYPs. The database can be accessed at http://p450.biodesign.ac.cn/.

#### Cytochrome P450Reaction Database

Zhang et al. (2024) created Cytochrome P450Reaction Database (P450Rdb), a specialized repository designed to catalogue P450-catalysed reactions documented in the scientific literature. This comprehensive database contains over 1,692 different reactions catalyzed by nearly 600 cytochrome P450 enzymes from more than 200 different species spanning the plant kingdom, animals, fungi, bacteria, and archaea. The curated reactions include 1,507 oxidations, 82 reductions, 61 catabolic processes, 29 combinatorial transformations, and a single substitution reaction.

P450Rdb serves as a valuable resource for differentiating between the myriad of plant CYP proteins and their respective reactions, providing detailed insights into the associated substrates, products, subcellular localization, and protein structures. However, P450Rdb's coverage of plant CYP sequences is somewhat limited and does not encompass the full spectrum of CYP types. In addition, the current coverage of reaction processes is modest and it does not yet have predictive capabilities for unknown CYP-mediated reactions. For those interested in exploring P450Rdb, the database can be accessed at https://www.cellknowledge.com.cn/p450rdb/index.html.

### Future Perspectives

Combining available gene copy features to assess gene family diversity and inferring motif conservation, along with individual copies, effectively contributes to a deeper understanding of the evolution of plant gene families. Among gene families that have been investigated, CYP stands out as the largest metabolic gene family, playing a broad and important role in various aspects of plant environmental adaptation, tissue and organ formation, and maintenance of secondary metabolism (Fernie et al. 2024; de Vries and Feussner 2024; Werck-Reichhart et al. 2024). Plant CYPs exhibit remarkable structural variation, with homology between different CYP sequences as low as 20% or less, largely owing to their diverse substrate and functional requirements. The differences in diversity, copy size, and structural conservation of CYPs among the 44 plant genomes examined in this study underscore the significant variation that exists between taxa. The conservation of similar CYPs within clans or families further supports the ancient status of CYP51, particularly the CYP51G subfamily, which is not found in other CYP families (Nelson et al. 2008). Additionally, the multiple copy sizes observed within the CYP51 family indicate that the formation of the CYP51H subfamily contributes to the overall increased copy size of the Monocotyledons 51 clan, making it the largest among plant lineages (Fang et al. 2021). Although clan 711 has traditionally been defined as a monophyletic clan, the presence of an ancient non-CYP711 family suggests an additional type of CYP diversity in Dicotyledoneae (Nelson et al. 2008). These findings suggest that clan 711 might be considered a potential polyphyletic clan, emphasizing the significant impact of changes in CYP family diversity on plant evolution. Interestingly, the Monocotyledons 711 clan consists of only a single CYP711 family, yet its copy size is higher, indicating a gradual loss of copies or the influence of other driving mechanisms on the copy evolution within this clan.

The significant copy size of clan 710 in Brassica, particularly in the diploid *Arabidopsis*, presents a contradiction to established theories of molecular evolution. Despite Brassica plants having a 1.4-fold larger copy size of the CYP gene, none of its close relatives exhibit a lower CYP710 copy size when compared to *Arabidopsis* (Yu et al. 2017). The reliability of this result requires further experimental evidence to explore potential limits to plant tolerance to CYP710 dosage, suggesting a potential bias in molecular evolutionary studies across genera. In contrast, clan 727, which is not commonly regarded as a core CYP family in angiosperms, exhibits a widespread distribution of CYP727 among various plant taxa, including from the beginning of gymnosperms. Although CYP727 loss may occur at different nodes, this suggests that the 10-lineage clan system could serve as a more convenient distribution system for angiosperm CYP diversity, contributing significantly to our understanding of plant metabolic diversity (Warren et al. 2015).

All CYPs share six conserved motifs that strongly contribute to the maintenance and formation of CYPases (Hasemann et al. 1995). These motifs include (P/I)PGPX(P/G)XP in the proline-rich region, WXXXR residues in the C-terminal helix, (A/G)GX(E/D)T(T/S) in the I-helix, the ERR triad (PERF residues in the electron transfer channel and EXXR residues in the K-helix), and the FXXGXXXCXG heme-binding loop (Chadha et al. 2018; Ershov et al. 2019; Zhang et al. 2020). However, the extensive expansion of CYPases in the plant kingdom may limit the applicability of these results. Our integrated evaluation suggests that plant CYPases may exhibit a significant number of variations, with abundant motif variations observed across different subfamilies, family levels, and species. These findings imply potential functional differences resulting from the functional diversification of plant CYP genes. Moreover, several plant CYP clans (clans 710, 727, 74, and 51) lack multiple motifs, highlighting the importance of structural differences in driving functional diversification in CYP research. Understanding the structural basis of plant CYP subfamilies or classifications is an important direction for future research.

## Supplementary Material

evae240_Supplementary_Data

## Data Availability

The data described in the article can be found in the main text or supplemental information.
