# Supplementary material for: Comprehensive Review on Plant Cytochrome P450 Evolution: Copy Number, Diversity, and Motif Analysis From Chlorophyta to Dicotyledoneae
Source: Genome Biol Evol. 2024 Nov 7;16(11):evae240. doi: 10.1093/gbe/evae240 (PMC11586672; doi:10.1093/gbe/evae240)
Supplement: evae240_Supplementary_Data [file evae240_supplementary_data.zip › Supporting Figures.docx]

**Comprehensive Review on Plant Cytochrome P450 Evolution: Copy Number, Diversity, and Motif Analysis from Chlorophyta to Dicotyledoneae**

Yuanpeng Fang, Zheng Tai, Keyi Hu, Lingfeng Luo, Sanwei Yang^*^, Mengmeng Liu^*^, Xin Xie^*^

College of Agriculture, Guizhou University, Guiyang 550025, PR China

* Corresponding authors:

E-mail address: [ippxiexin@163.com](mailto:ippxiexin@163.com) (X. Xie); swyang@gzu.edu.cn (S. Yang); liumengmeng1006@126.com (M. Liu)

**SUPPORTING INFORMATION**

**SUPPORTING FIGURES**

**
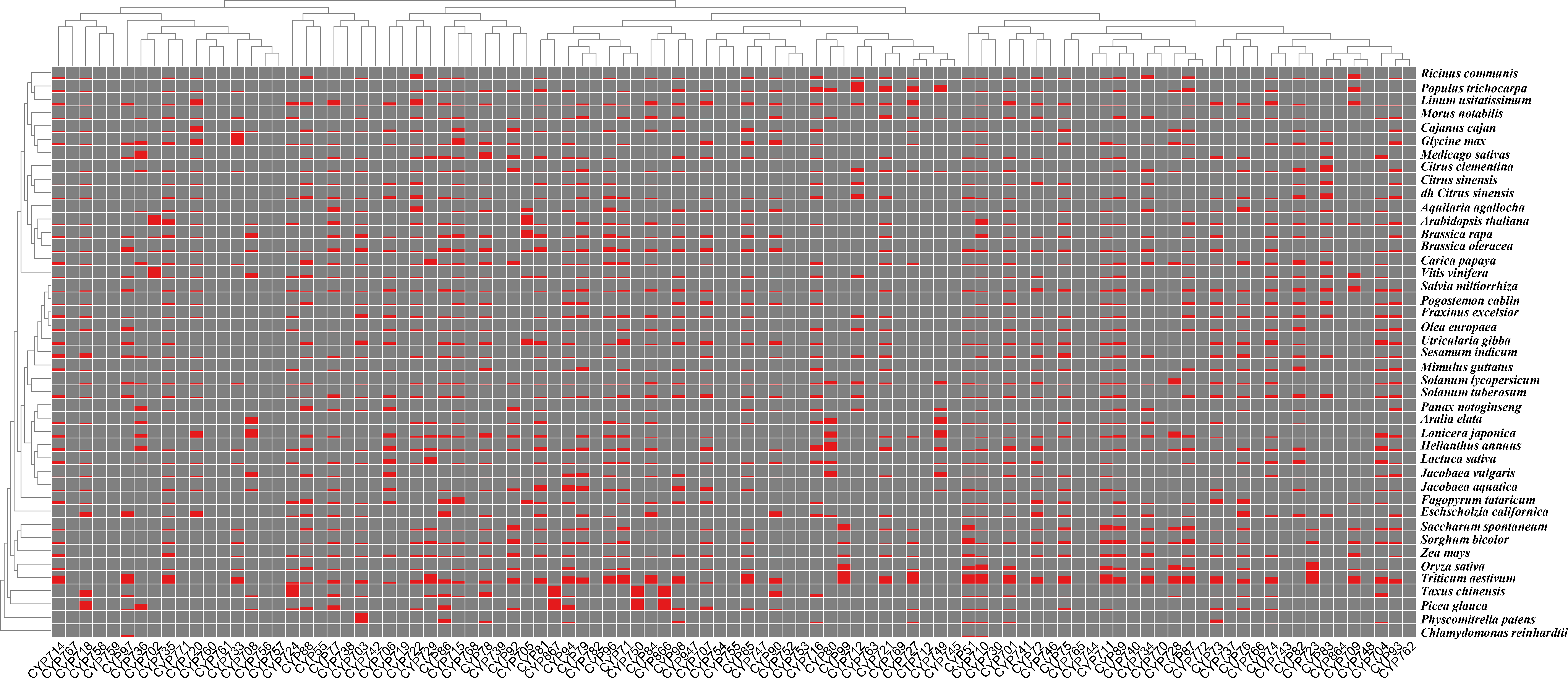
**

**Figure S1** Heat map of number distribution of the cytochrome P450 (CYP) gene family in identified plants.


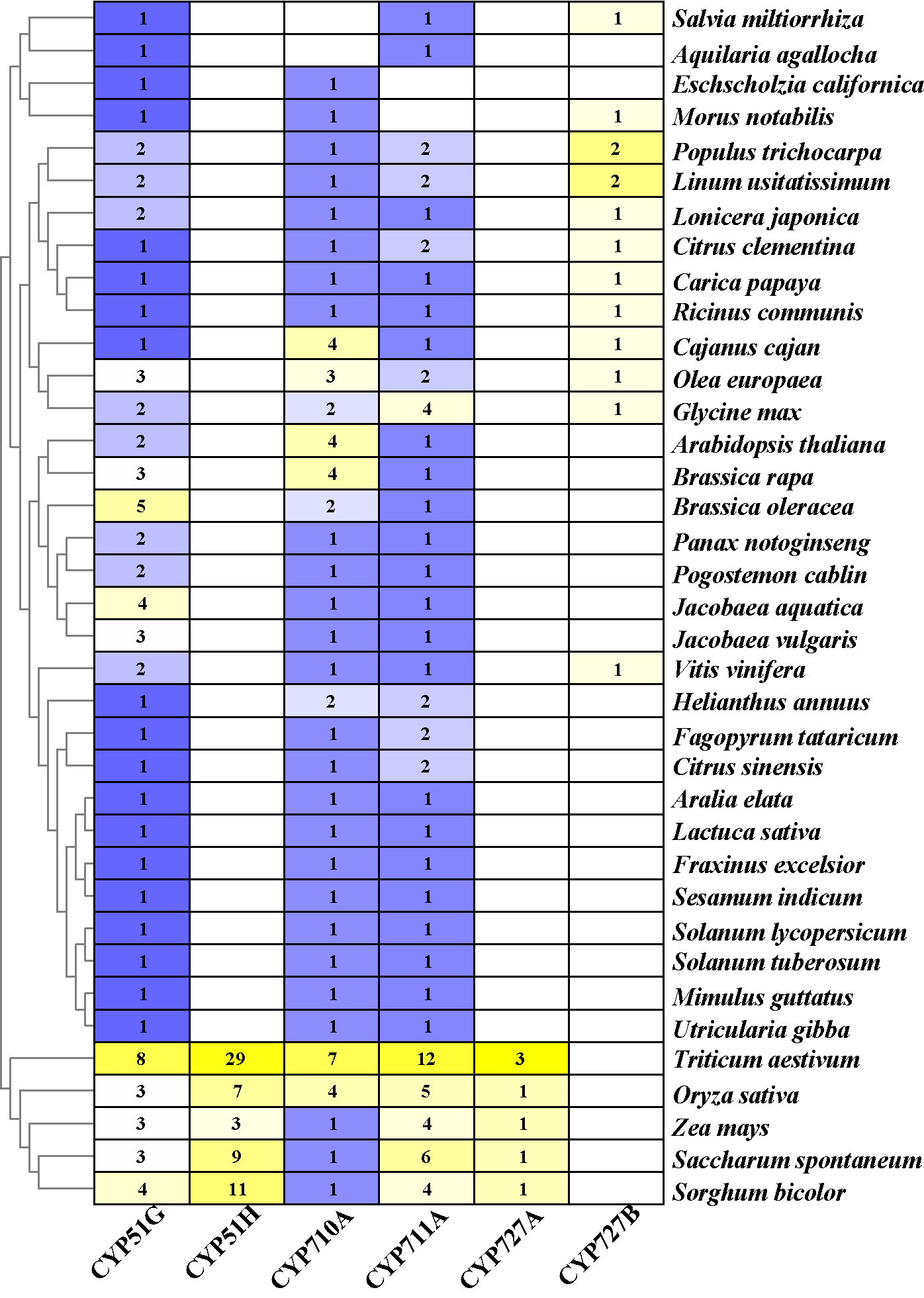


**Figure S2** Gene copy number of CYP51, CYP711, CYP710, and CYP727 subfamilies in angiosperms


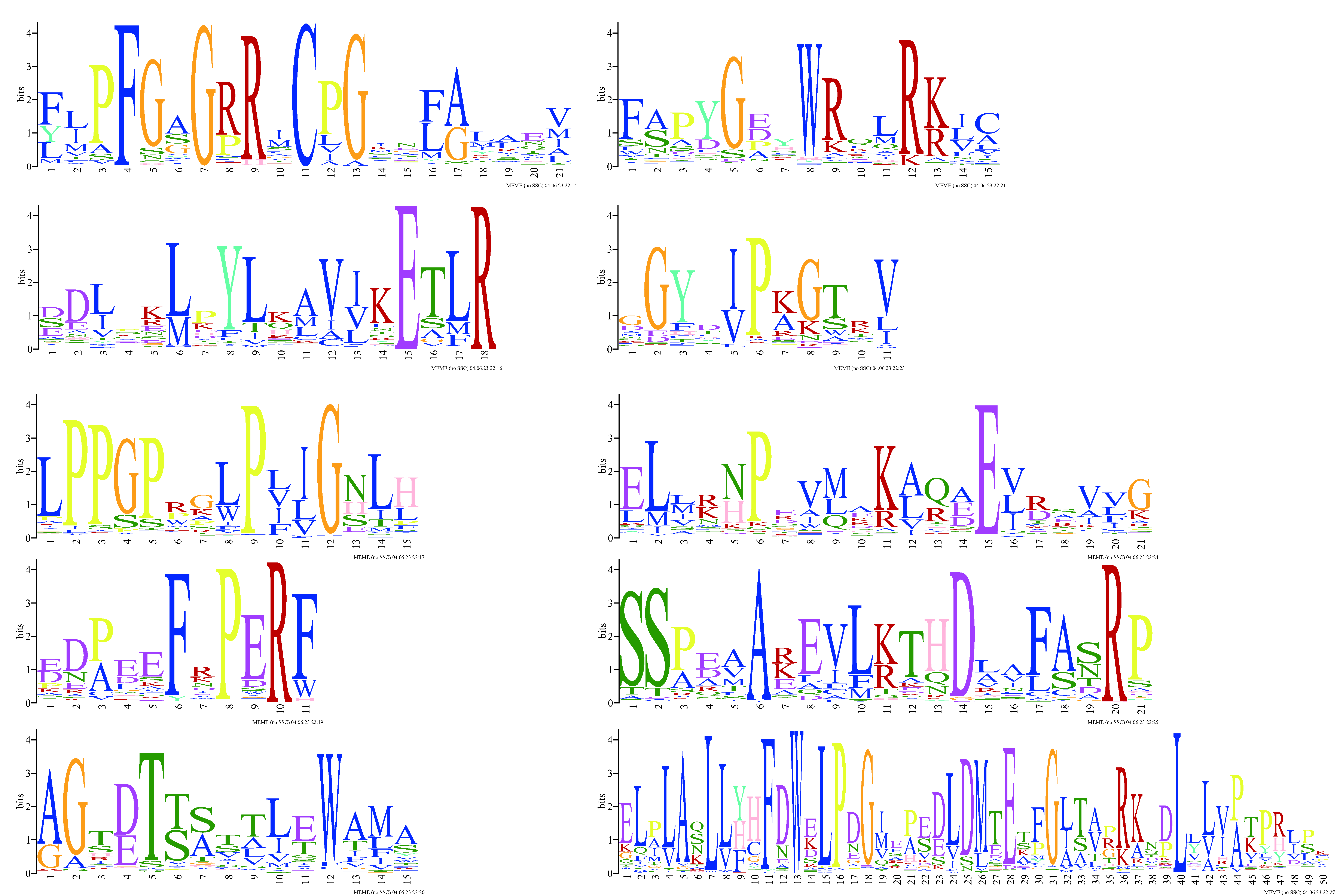


**Figure S3** The logo of the 10 most conserved motifs in plant cytochrome P450 enzymes (CYPs)


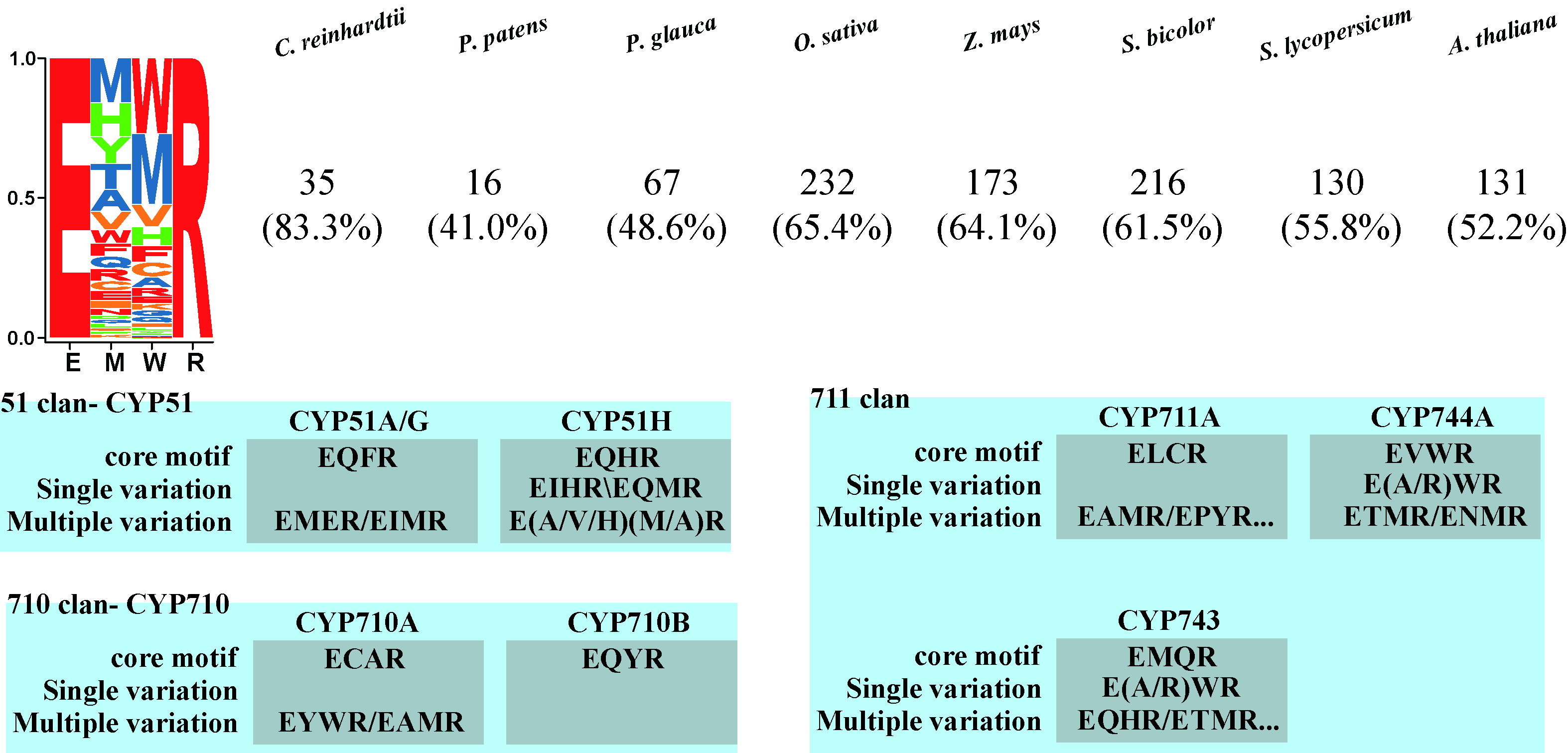


**Figure S4** Logo of EXXR motifs in plants, proportion of conserved motif proteins, and site variations in the cytochrome P450 (CYP) family of single lines

**
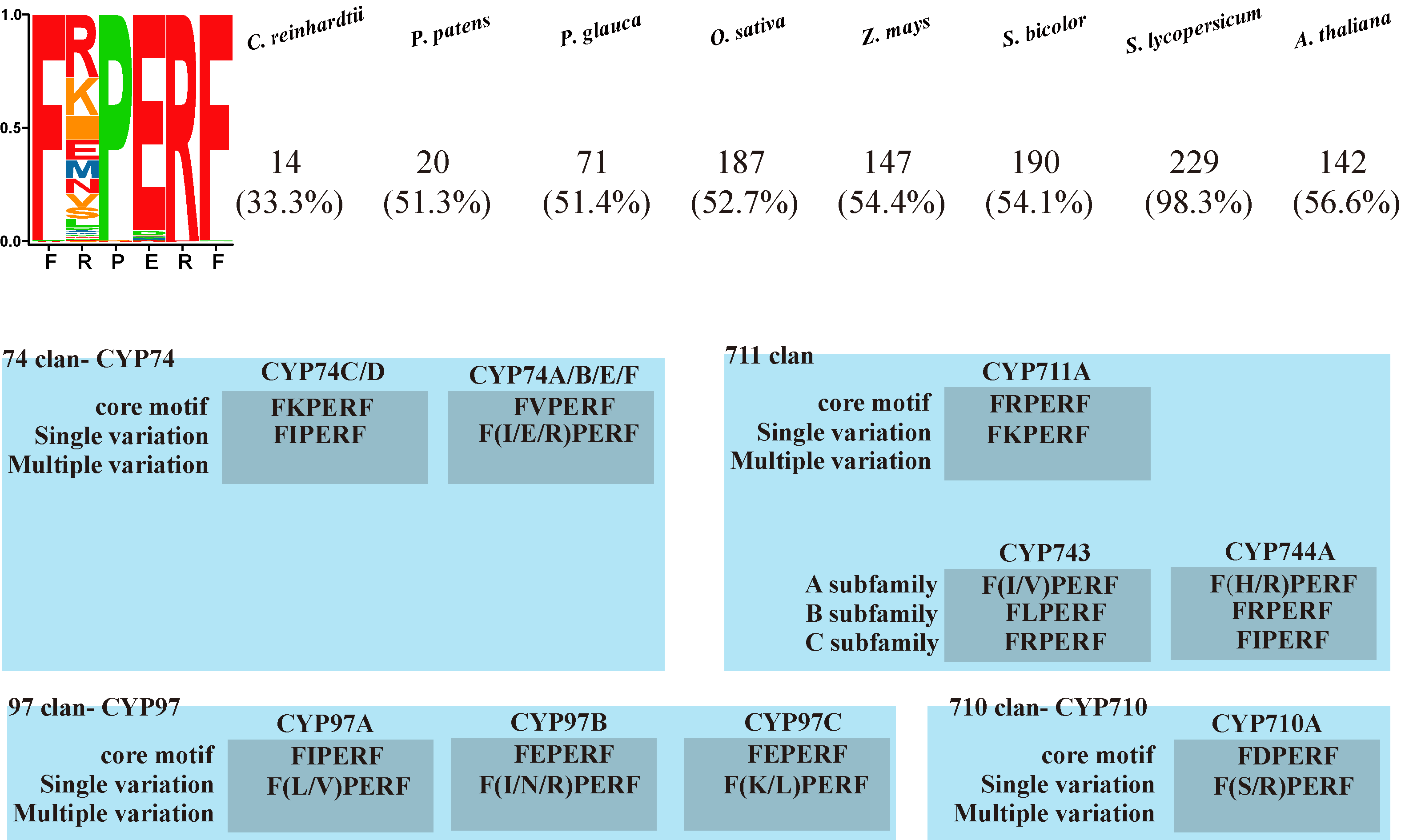
**

**Figure S5** Logo of FXPERF motifs in plants, proportion of conserved motif proteins, and site variations in the cytochrome P450 (CYP) family of single lines

**
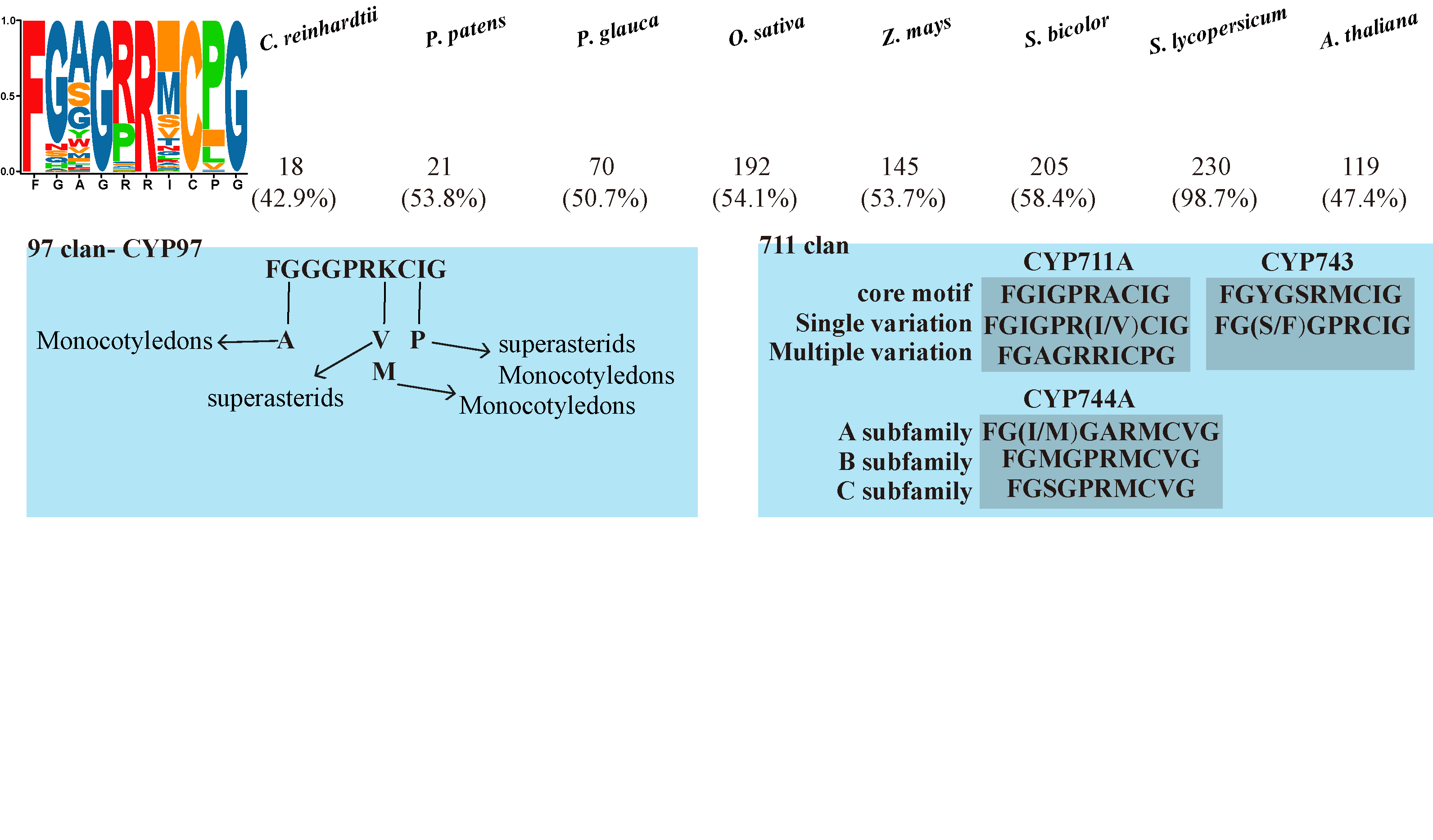
**

**Figure S6** Logo of FXXGXRXCXG motifs in plants, proportion of conserved motif proteins, and site variations in the cytochrome P450 (CYP) family of single lines

**
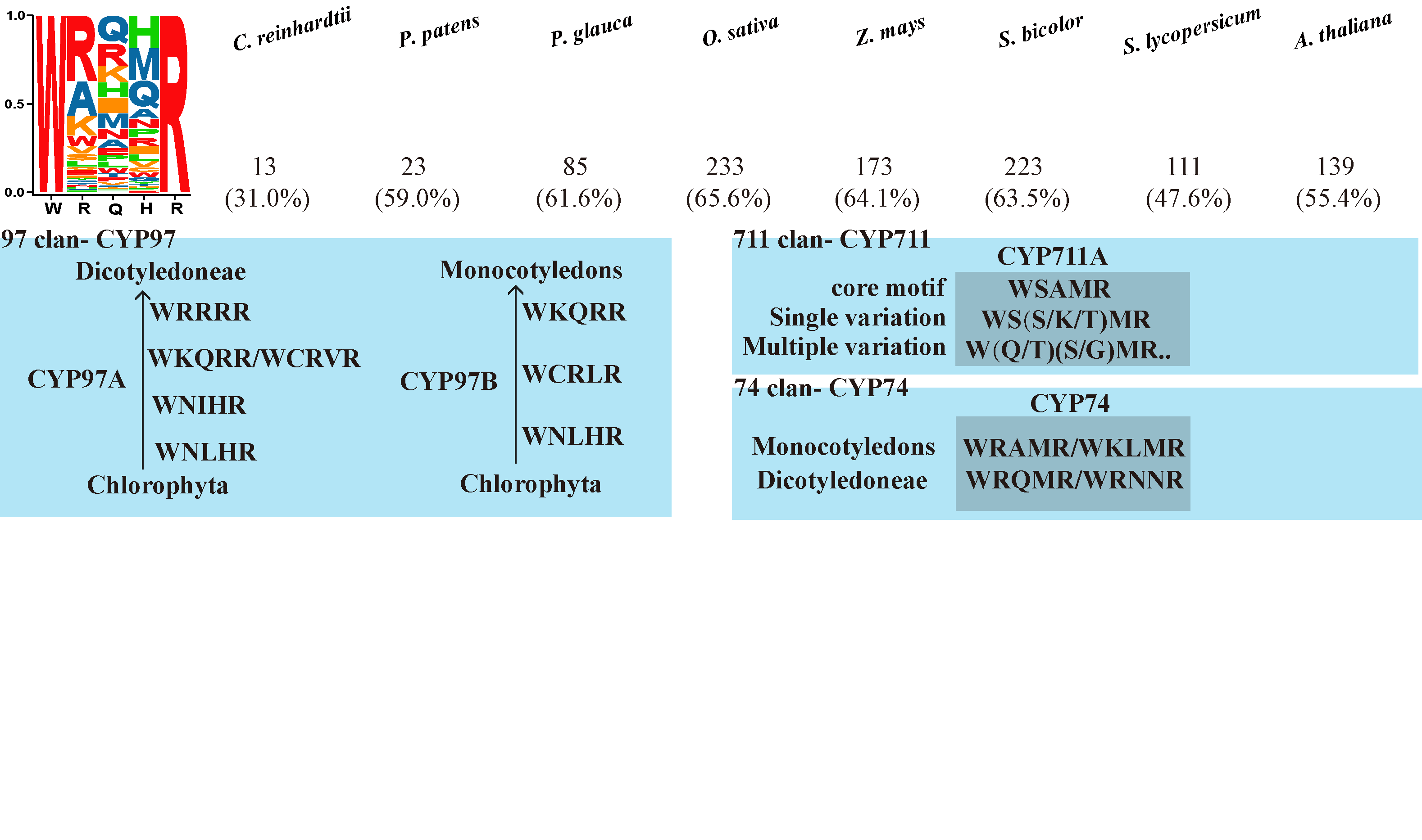
**

**Figure S7** Logo of WXXXR motifs in plants, proportion of conserved motif proteins, and site variations in the cytochrome P450 (CYP) family of single lines


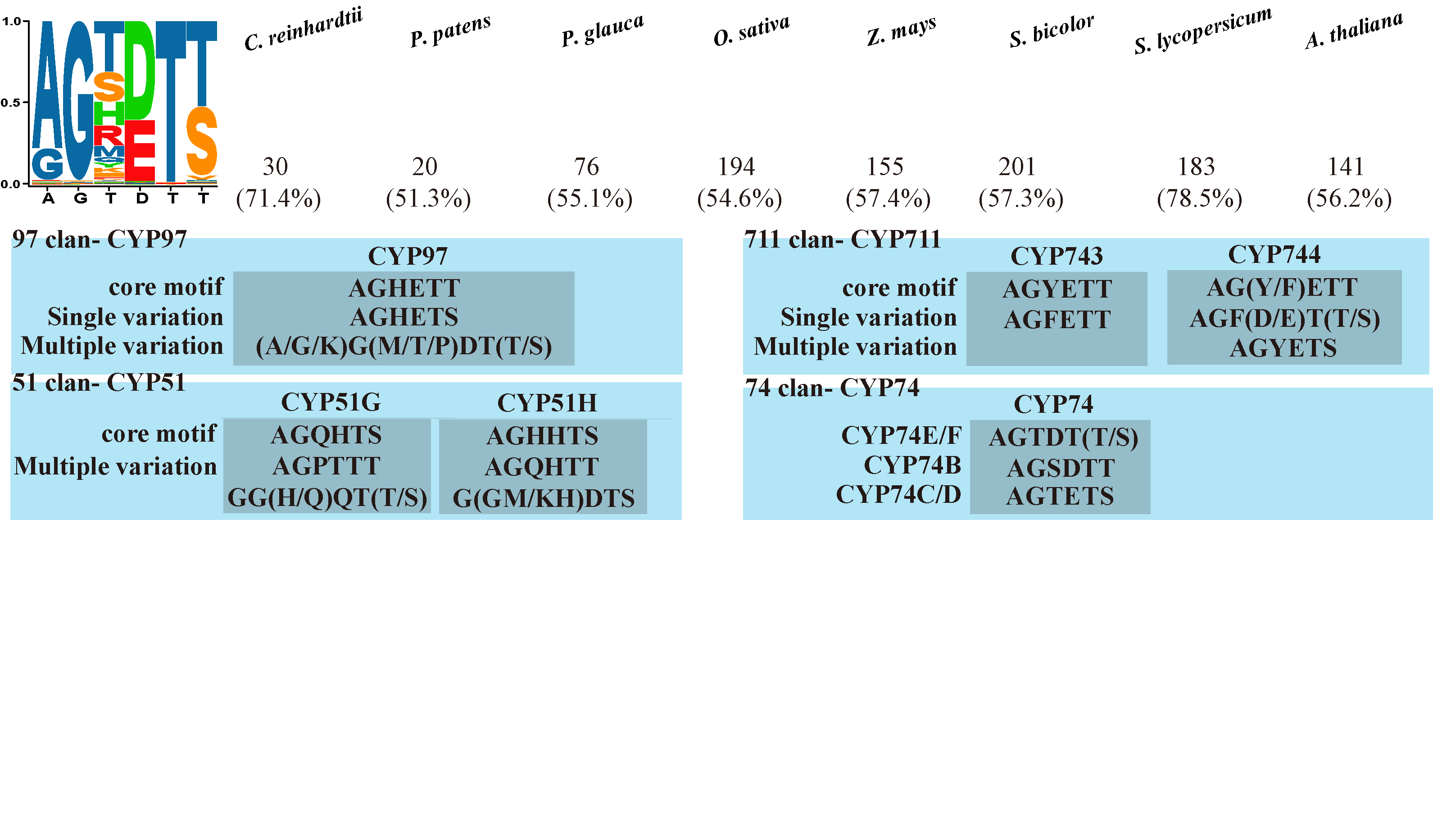


**Figure S8** Logo of (A/G) GX (D/E) T (T/S) motifs in plants, proportion of conserved motif proteins, and site variations in the cytochrome P450 (CYP) family of single lines

**
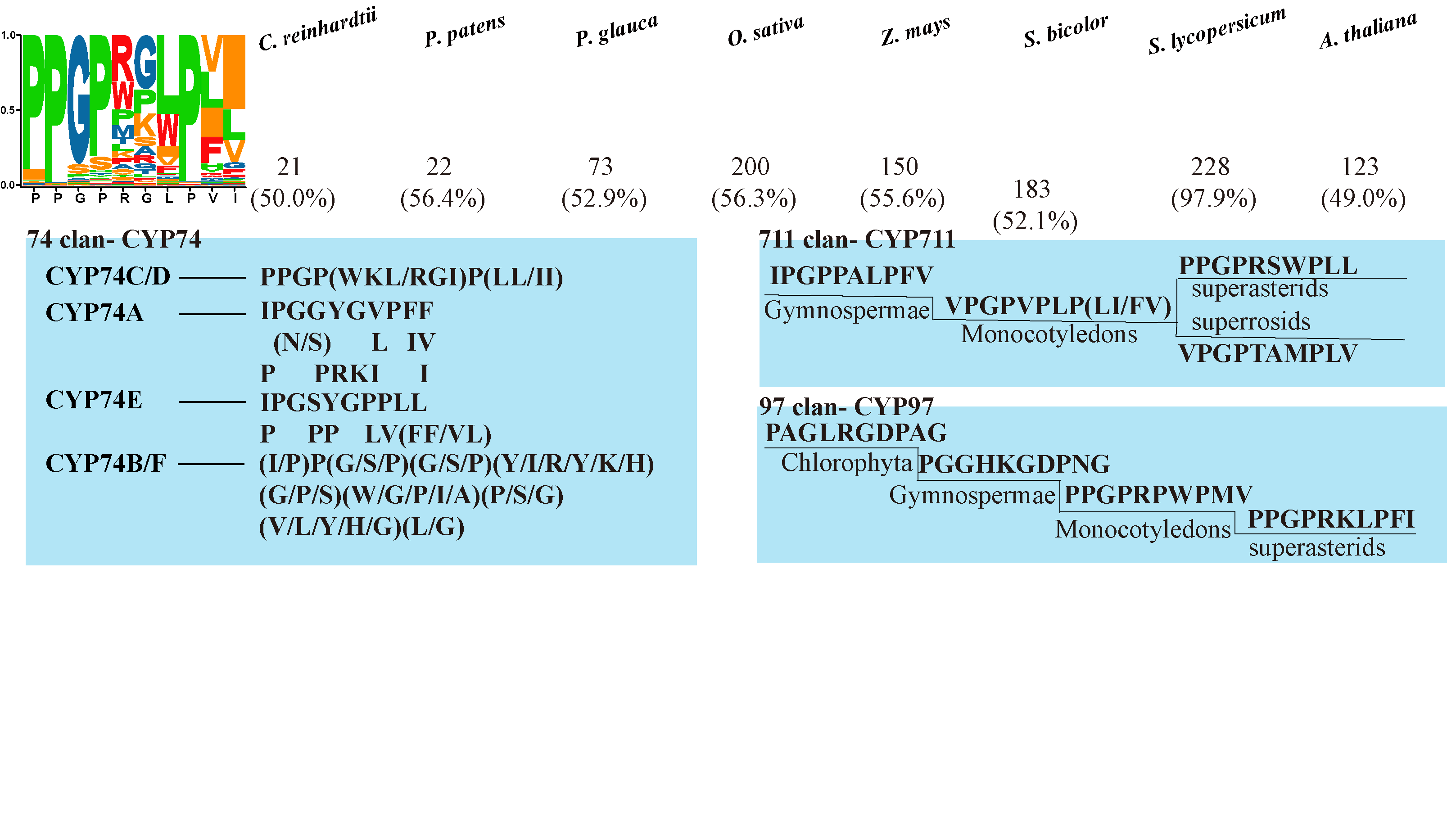
**

**Figure S9** Logo of (P/I) PGPX (P/G) XP motifs in plants, proportion of conserved motif proteins, and site variations in the cytochrome P450 (CYP) family of single lines
